# Galectin-3 is associated with a poor prognosis in primary hepatocellular carcinoma

**DOI:** 10.1186/s12967-014-0273-3

**Published:** 2014-09-27

**Authors:** Shan-Shan Jiang, De-Sheng Weng, Qi-Jing Wang, Ke Pan, Yao-Jun Zhang, Yong-Qiang Li, Jian-Jun Li, Jing-Jing Zhao, Jia He, Lin Lv, Qiu-Zhong Pan, Jian-Chuan Xia

**Affiliations:** State Key Laboratory of Oncology in Southern China and Department of Experimental Research, Sun Yat-sen University Cancer Center, 651 Dongfeng Road East, Guangzhou, 510060 PR China; Department of Hepatobiliary Oncology, Sun Yat-sen University Cancer Center, Guangzhou, People’s Republic of China

**Keywords:** Galectin-3, Hepatocellular carcinoma, Angiogenesis, Apoptosis, Prognostic factor

## Abstract

**Background:**

Galectin-3, a member of the beta-galactoside-binding lectin family, is a multifunctional protein with various biological functions, including the proliferation and differentiation of tumor cells, angiogenesis, cancer progression, and metastasis. We aimed to clarify if expression of galectin-3 is related to the clinicopathological characteristics and prognosis of hepatocellular carcinoma (HCC) patients, and to explore the possible mechanisms of galectin-3 in hepatocellular carcinoma.

**Methods:**

First, we investigated galectin-3 mRNA and protein expression by using RT-PCR and Western blotting. Second, tissues from 165 HCC patients were used to evaluate clinicopathological characteristics and prognosis through immunohistochemical analyses. Furthermore, the functions of galectin-3 were analyzed with respect to the proliferation, cell cycle,apoptosis, migration, and invasion of HCC cell lines. Finally, we analyzed galectin-3 expression and micro-vessel density (MVD) by immunohistochemistry (IHC) to find its correlation with angiogenesis in Hepatocellular Carcinoma. Flow cytometer was used to explore apoptosis and Western-blot was used to detect the pathway proteins of apoptosis.

**Results:**

Galectin-3 showed high expression at the mRNA and protein levels in HCC cancer tissues and cell lines. Clinicopathological analyses revealed that increased expression of galectin-3 in tumors was closely associated with a poor prognosis. Galectin-3 knockdown by siRNA significantly inhibited cell growth, migration, and invasion, and induced apoptosis in HCC cells in vitro, whereas galectin-3 overexpression promoted cell growth, migration, and invasion. Correlation analysis of galectin-3 expression and micro-vessel density (MVD) showed that galectin-3 expression in tumor cells stimulates angiogenesis. The observed regulation of cell apoptosis was accompanied by the galectin-3-mediated modulation of caspase3 signaling pathways in HCC cells.

**Conclusions:**

These data suggest that galectin-3 plays an important part in HCC progression and may serve as a prognostic factor for HCC.

## Introduction

Hepatocellular carcinoma (HCC) is one of the most prevalent and lethal malignancies worldwide. HCC is the second most frequent cause of cancer deaths and the fifth most commonly diagnosed cancer in the world, and is particularly prevalent in southeastern Asia and sub-Saharan Africa [1,2]. Options for curative treatment for early-stage HCC are surgery, transarterial chemoembolization (TACE), chemotherapy, biotherapy and radiotherapy [3]. The outcome and prognosis of HCC are disappointing. Only 10–20% of tumors are resectable at the time of diagnosis, and the five-year survival is poor even compared with other gastrointestinal malignancies [4]. Hepatocarcinogenesis is a multifactorial and multistep process that involves activating oncogenes and inactivating tumor suppressor genes at different stages during the progression of HCC [5-7].

The galectins are a family of beta-galactoside-binding proteins implicated in the modulation of cell–cell and cell–matrix interactions [8,9]. Fifteen galectins have been identified in mammals. Galectin-3 is expressed widely and is structurally unique. It comprises three distinct domains: a short NH_2_-terminal domain containing a serine phosphorylation site; a repeated collagen-like sequence; and a COOH-terminal domain containing a single carbohydrate recognition-binding domain [10,11]. Galectin-3 is a multifunctional protein implicated in various biological functions, including: the adhesion, proliferation and differentiation of tumor cells, angiogenesis, cancer progression and metastasis [12]. Cytosolic galectin-3 is targeted to the plasma membrane and released into the extracellular space, where it participates in the regulation of the migration and adhesion of cells [13]. Galectin-3 is also targeted to the nucleus, where it plays a part in pre-mRNA splicing and activation of diverse transcription factors [14]. Galectin-3 has been found to be correlated to several cancers, including mesothelioma [15] as well as cancer of breast [16], gastrointestinal system [17], and colon [18]. Shimosegawa et al. [19] reported that in HCC, galectin-3 expression was correlated with histological differentiation and vascular invasion, and that patients who expressed galectin-3 tended to relapse earlier and had poorer overall survival. However, the number of cases in their study was relatively small (52 patients). Hence, the function of galectin-3 in HCC has not been fully characterized.

We aimed to evaluate the correlations between the clinicopathological features and galectin-3 expression in a larger group of Chinese HCC patients (165 cases). We also attempted to analyze the prognostic value of galectin-3 expression. Moreover, we assessed the effect of galectin-3 on the progression of HCC by assessing the effect of knockdown and overexpression of galectin-3 on the proliferation, migration, invasion, cell cycle and apoptosis in HCC cells in vitro.

## Materials and methods

### Ethical approval of the study protocol

This study was approved by the Ethics Committee of the Cancer Center of Sun Yat-sen University (Guangdong, China). Written informed consent was obtained from each patient.

### Cell culture

The human HCC cell lines, HepG2 (well differentiated, low metastatic potential), Hep3B (well differentiated, low metastatic potential), and Human liver adenocarc -inoma Endothelial cell line, Sk-Hep1 were obtained from the American Type Culture Collection (Manassas, VA, USA). Huh7 cells (well differentiated, low metastatic potential) were obtained from the Riken Cell Bank (Ibaraki, Japan). Bel-7402 cells (moderate differentiated, low metastatic potential) and the normal liver cell line LO2 were obtained from the Committee of Type Culture Collection of the Chinese Academy of Sciences (Shanghai, China). All cells were cultured in RPMI 1640 supplemented with 10% fetal bovine serum (FBS) in 5% CO_2_ at 37°C.

### Patients and tumor tissue samples

Tissue samples, including HCC tumor tissues and adjacent non-cancerous tissues (n = 44), were obtained from patients who had resection of primary HCC in the Cancer Center of Sun Yat-sen University between 2011 and 2012. None of these patients had received preoperative chemotherapy or radiotherapy. After resection, matched fresh tissues were immersed immediately in RNAlater® (Ambion, Austin, TX, USA), kept overnight at 4°C, then stored at −80°C until RNA isolation. A total of 165 paraffin-embedded HCC samples were obtained from patients who underwent hepatectomy at the Cancer Center of Sun Yat-sen University between 2001 and 2004. Follow-up was in our outpatient department, and involved clinical and laboratory examinations every 3 months for the first 2 years, every 6 months during the third to fifth years, and annually for an additional 5 years or until death, whichever occurred first. Overall survival (i.e., time from surgery to death or final follow-up) was used as a measure of prognosis. Histological types were assigned according to classification criteria set by the World Health Organization.

### RNA preparation and protein extraction

Total RNA was extracted by using Trizol solution (Invitrogen, Shanghai, China) according to manufacturer’s instructions. Total protein was extracted with RIPA buffer (Beyotime, Shanghai, China) according to the manufacturer’s protocol. RNA and protein samples were stored at −80°C until use.

### Real-time quantitative reverse transcription-polymerase chain reaction (RT-PC R)

Real-time PCR amplification was undertaken with an ABI 7900HT Real-time PCR system (Life Technologies, Carlsbad, CA, USA). The primers used for amplifying galectin-3, caspase3, caspase9, PARP, BAX, Bcl-2, GAPDH were selected. That is, for galectin-3, they were: forward, 5′-ACGAGCGGAAAATGGCAGA-3′; and reverse, 5′-GATAGGAAGCCCCTGGGTAGC-3′. For glyceraldehyde-3-phosphatedehydroge nase (GAPDH), forward 5′-CTCCTCCTGTTCGACAGTCAGC-3′, and reverse, 5′-CCCAATACGACCAAATCCGTT-3; for caspase3, forward 5′-ATCTCGG TCTG GTACAGATGTCGAT-3′, and reverse 5′-TGAATTTCGCCAAGAATAATACCA-3′; for caspase9, forward 5′-GCCATGGACGAAGCGGATCGGC-3′, reverse 5′-GGC CTGGATGAAGAAGAGCTTGGG-3′; for PARP, forward primer 5′-CGGAGTCTT CGGATAAGCTCT-3′, reverse primer 5′-TTTCCATCAAACATGG GCGAC-3′; for BAX, forward primer 5′-CCCGAGAGGTCTTTTTCCGAG-3′, reverse primer 5′-CCAGCCCATGATGGTTCTGAT-3′; for Bcl-2, forward primer 5′-GGTGGGGTCA TGTGTGTGG-3′, reverse primer 5′-CGGTTCAGGTACTCAGTCATCC-3′. The PCR was carried out in a final volume of 15 μL, consisting of 7.5 μL of 2× SYBR Green master mix (Invitrogen), 2 μL of each 5′- and 3′- primer (1.5 pmol/μL), 0.5 μL of the sample cDNA, and 5 μL water. The PCR conditions were 95°C for 10 min, one cycle, followed by 95°C for 30 s and 60°C for 60 s, 45 cycles. The relative expression levels of galectin-3, caspase3, caspase9, PARP, BAX, Bcl-2 were normalized to that of the internal control gene, GAPDH. Data were analyzed using the comparative threshold cycle (2– –ΔΔCT) method.

### Immunohistochemistry

Isolated tumors were fixed in 10% neutral buffered formalin for 48 h and embedded in paraffin according to standard protocols. Sections (thickness, 4 μm) were deparaffinized and rehydrated in a graded series of alcohol solutions. For antigen retrieval, slides were immersed in ethylenediamine tetra-acetic acid (EDTA; 1 mmol/L, pH8.0) and boiled for 15 min in a microwave oven. Endogenous peroxidase acti- vity was blocked in 3% H_2_O_2_ at room temperature for 15 min, and non-specific binding was abolished by 5% bovine serum albumin (BSA) for 30 min. Sections were then stained with anti-galectin-3 (rabbit anti-galectin-3 polyclonal antibody; 1:250 dilution; Abcam, Cambridge, UK) antibody, anti-CD34 (rabbit anti-CD34 polyclonal antibody; 1:400 dilution; Gene Tech, Shanghai, China) antibody at 4°C overnight. After washing with phosphate-buffered saline (PBS), sections were incubated with horseradish peroxidase (HRP)-conjugated secondary antibody (Envision Detection kit, GK500705, Gene Tech, Shanghai, China) at room temperature for 30 min. After washing thrice with PBS, antibody complexes were colored with 3, 3′-diamino benzidine and then counterstained with hematoxylin. Slides were dehydrated and evaluated.

### Semi-quantitative method

The total galectin-3 immunostaining score was calculated as the sum of the positively stained tumor cells and staining intensity. Briefly, the percentage of positive staining was scored as “0” (<5%, negative), “1” (5–25%, sporadic), “2” (25–50%, focal), or “3” (>50%, diffuse). Staining intensity was scored as “0” (no staining), “1” (weak staining), “2” (moderate staining), or “3” (strong staining). Both the percentage of positive cells and the staining intensity were evaluated under double-blind conditions. The total immunostaining score was calculated as the value of percent positivity score × staining intensity score, and ranged from 0 to 9. We defined galectin-3 expression levels as: “–” (score 0–1), “+” (2–3), “++” (4–6) and “+++” (>6). Based on their levels of galectin-3 expression, patients were divided into two groups: low galectin-3 (“–” and “+”) and high galectin-3 (“++” and “+++”). For intratumoral MVD assessment, micro-vessels were recorded by counting CD34-positive stained endothelial cells according to the international consensus on the methodology and criteria of evaluation of angiogenesis quantification in solid tumors. After scanning the whole section at low magnifications (100×), ten tumor areas with the greatest number of distinctly highlighted micro-vessels (hot spot) were selected. The value of MVD was evaluated by the average of ten 200 × field micro-vessel counts. Data were shown as mean ± SD. The score assessment was performed independently by two pathologists blinded to the clinical parameters.

### Western blotting

HCC samples (tumor and adjacent non-tumor tissues) and cell lines were lysed in RIPA lysis buffer. Lysates were harvested by centrifugation (12,000 rpm for 20 min at 4°C. Protein samples (≈30 μg) were resolved in 12% sodium dodecyl sulfate polyacrylamide gel for electrophoresis and transferred to a polyvinylidene difluoride (PVDF) membrane. After blocking non-specific binding sites for 60 min with 8% non-fat milk, membranes were incubated overnight at 4°C with a rabbit polyclonal antibody against galectin-3 (1:1,000 dilution; Abcam), caspase3 (1:500 dilution, cell signaling technology), caspase9 (1:500 dilution, cell signaling technology), PARP (1:500 dilution, cell signaling technology), BAX (1:500 dilution; Proteintech Group), Bcl-2 (1:1000 dilution; Proteintech Group) or GAPDH (1:10,000 dilution; Proteintech Group). Membranes were washed four times with TRIS-buffered saline with Tween-20 for 10 min. After washing, membranes were probed with HRP-conjugated secondary antibody and visualized using a chemiluminecent system (Cell Signaling Technology, Danvers, MA, USA). Band intensity was measured by Quantity One software (BioRad, Hercules, CA, USA).

### RNA oligonucleotides and cell transfection

For transient transfection experiments, certain small interfering ribonucleic acid (siRNA) molecules were used. That is, for galectin-3-siRNA: sense, 5′-GUACA AUCAUCGGGUUAAATT-3′ and antisense, 5′-UUUAACCCGAUGAUUGUACTT-3′. For the negative control: sense, 5′-UUCUCCGAACGUGUCACGUTT-3′ and anti- sense, 5′-ACGUGACACGUUCGGAGAATT-3′. The siRNAs were synthesized by GenePharma (Shanghai, China). Cells at around 70% confluence were transfected with the indicated siRNA using Lipofectamine RNAiMax reagent (Invitrogen) according to manufacturer’s instruction. 72 hours after transfection, cells were detached with trypsin/EDTA, suspension, and allowed to grow overnight before treatment. Knockdown efficiency was evaluated by western blotting.

### Recombinant Lentivirus vector construction and tumor cell infection

The galectin-3-overexpressing recombined Lentivirus vector and the control vector were constructed by GenePharma (Shanghai, China). Lentiviral infection was performed by adding virus solution to Hep3B cells in the presence of 5 μg/ml polybrene (Sigma-Aldrich, St. Louis, MO, USA). After infection for 48 h, the cells were selected in the presence of 2 μg/ml puromycin, and puromycin-resistant cells were collected and cultured. The stable cell lines were specified as Hep3B-galectin-3 and Hep3B-mock, respectively.

### Cell proliferation assay

Cell growth rates were measured with a (3-(4, 5-dimethylthiazol-2-yl) -5-(3-carboxy methoxyphenyl)-2-(4-sulfophenyl)-2H-tetr-azolium) (MTS) cell proliferation assay. Cells were plated in triplicate in 96-well plates at 2500 cells per well. At each time point, the medium was removed and cells incubated with 20 μL of MTS (5 mg/mL; Sigma-Aldrich, St Louis, MO, USA) for 4 hours in 5% CO_2_ at 37°C. Finally, the optical density of formazan was measured using a microplate reader at 490 nm. Three independent experiments were performed to analyze the cell growth. Statistical analyses were carried out using the two-tailed unpaired Student’s *t*-test.

### Cell cycle assay

HCC cells transfected with gal-siRNA, NC, galectin-3-overexpression were collected after 36 h, washed twice in PBS, and fixed in 75% ethanol overnight at −20°C. Fixed cells were washed with ice-cold PBS once, resuspended in 500 μL PBS and 20 μL RNaseA, and then incubated in a 37°C water-bath for 30 min. Cells were stained with propidium iodide (PI; Bestbio, Shanghai, China) at 4°C in the dark for 30–60 min. Flow cytometry data were analyzed using Beckman Coulter (Fullerton, CA, USA) software.

### Apoptosis assay

HCC cells transfected with gal-siRNA, NC, galectin-3-overexpression were collected after 72 h. This was followed by trypsinization, centrifugation and washing with ice-cold PBS twice. Cells were then resuspended in 400 μL 1× binding buffer, incubated with 5 μL AnnexinV- fluorescein isothiocyanate and 10 μL PI for 15 min in the dark at 2–8°C. The numbers of stained cells were analyzed by a flow cytometer (Beckman Coulter). Each experiment was conducted in triplicate. Statistical analyses were carried out using the two-tailed unpaired Student’s *t*-test.

### Matrigel invasion assay

Invasion assays were carried out using transwell membrane filter inserts (diameter, 6.5 mm; pore size, 8 μm) in a 24-well tissue culture plate. Briefly, transfected Bel-7402, HepG2, Huh7 and Hep3B cells were harvested at 24 h and resuspended in serum-free RIPM 1640. Cells (1 × 10^5^/well) in 200 μL of growth medium without FBS were added to the upper chamber, and the bottom chamber was filled with 500 μL of growth medium containing 10% FBS. After 48 h, non-migrating cells were removed from the top of the filter with a cotton swab. Invading cells on the bottom of the filter were fixed with methanol, stained with 0.5% crystal violet, and counted. Stained cells at 10 random fields were counted using an inverted microscope. Each experiment was conducted in triplicate. Statistical analyses were carried out using the two-tailed unpaired Student’s *t*-test.

### Wound-healing assay

We assessed the migration of control- and galectin-3-siRNA, galectin-3-overex -pression transfected cells using an in vitro wound-healing assay. HepG2, Bel-7402, Hep3B cells (1 × 10^5^cells/well) transfected with gal-siRNA, galectin-3-overexpression and negative control were plated in six-well plates, wounded by scratching with a pipette tip, then incubated with RIPM 1640 without 10% FBS for 24 h in 5% CO_2_ at 37°C. All experiments were carried out in triplicate.

### Cell migration assay

The cell migration assays were performed in a chamber system consisting of polycarbonate membrane inserts with an 8-μm pore size (Corning, USA) placed in 24-well cell culture insert companion plates. The migration assay was conducted at 48 hours after the HepG2, Bel-7402, Hep3B and Huh7 cells were infected with siRNA, negative control, and galectin-3-overexpression. The cells (in 200 μL of growth medium without FBS) were placed in the upper chamber and 500 μL of growth medium with 5% FBS was placed in the lower chamber. The cells were incubated at 37°C for 24 hours. Following the incubation, the insert membranes were fixed with 75% methanol for 30 minutes, stained with 0.5% crystal violet, and counted. The stained cells were counted under an inverted microscope (10 fields per membrane). Each experiment was performed in triplicate. Statistical analyses were carried out using the two-tailed unpaired Student’s *t*-test.

### Statistical analyses

Statistical analyses were conducted using SPSS version 17.0 (SPSS, Chicago, IL, USA). The paired-samples *t*-test was used to investigate the differences in expression of mRNA and protein in HCC tumors and non-tumor tissue samples. The correlation between galectin-3 expression and clinicopathological features was evaluated by the χ^2^ test. Overall survival was calculated using the Kaplan–Meier method and the log-rank test was used for comparison. The Cox proportional hazards regression model was used for univariate and multivariate analyses. The results were expressed as mean ± SD and analyzed using the Students’ t-test. Differences were considered significant at p < 0.05.

## Results

### Galectin-3 mRNA and protein expression in HCC

**Figure 1:**
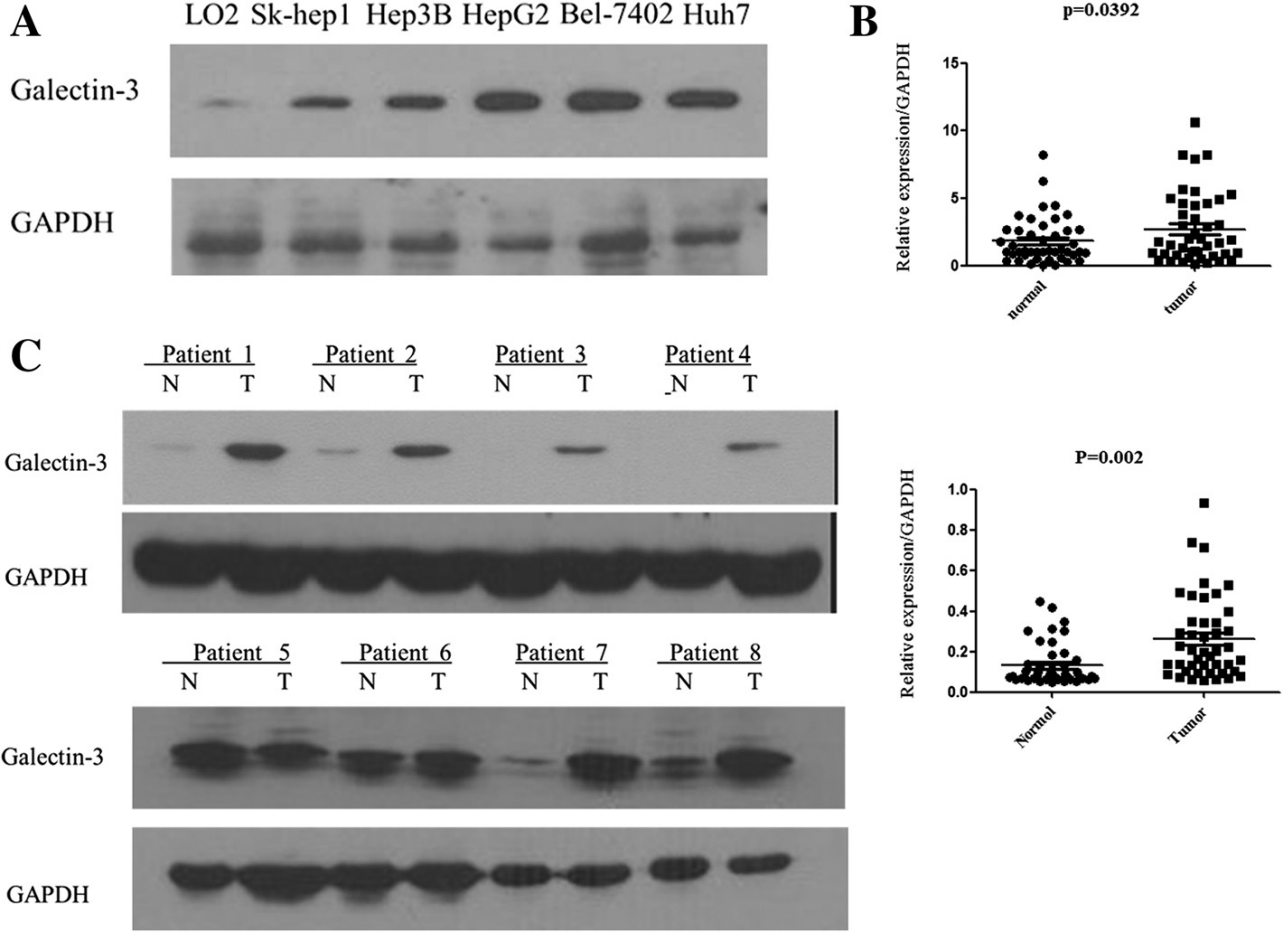
**Expression of galectin-3 mRNA and protein in human primary HCC cell lines and tissues as evaluated by real-time quantitative PCR and Western blotting. (A)** The protein transcript levels of galectin-3 in the Hep3B, Huh7, HepG2, and Bel7402 HCC cells were higher than in the normal liver cell line LO2. **(B)** The relative mRNA expression of galectin-3 was higher in 44 HCC tumor tissues than in adjacent non-tumor tissues (p = 0.039). **(C)** Galectin-3 protein expression was increased in tumor tissues compared with adjacent non-tumorous tissues (p = 0.002).

Firstly, HCC cell lines and primary HCC tissue samples were used to examine galectin-3 expression by quantitative real-time PCR and Western blot analyses. Western blot analyses confirmed that the protein transcript levels of galectin-3 in all four HCC cell lines were higher than the normal liver cell line LO2 (Figure 1A). Additionally, in 44 paired primary HCC tissue samples, an increased mRNA level of galectin-3 was observed in most tumor tissues (28/44) compared with the corresponding adjacent non-tumor tissues, as evidenced by quantitative real-time PCR analyses (*P* = 0.039; Figure 1B). This tendency was also verified at the protein level with western blot analyses (*P* = 0.002; Figure 1C).

### Association of galectin-3 with the clinicopathological features of HCC

**Figure 2:**
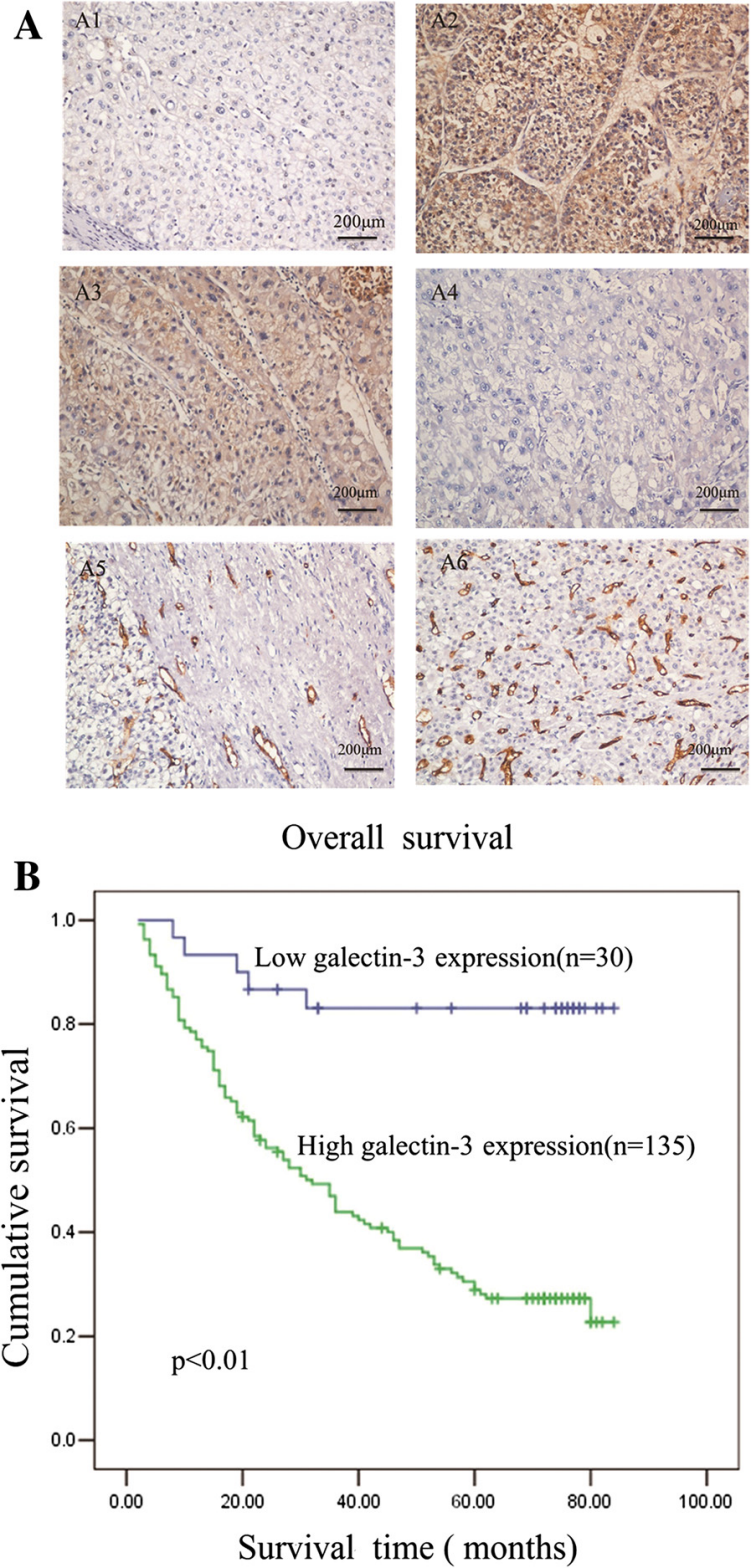
**Decreased galectin-3 and CD34 expression in surgical specimens of primary hepatocellular carcinoma (n = 165) detected by immunohistochemistry.** **(A1-4)** Immunohistochemical staining for galectin-3 expression in human hepatocellular carcinoma. (A1) Adjacent non-tumorous tissues. (A2) Poorly differentiated hepatocellular carcinoma. (A3) Moderately differentiated hepatocellular carcinoma. (A4) Well-differentiated hepatocellular carcinoma. (A5-6) Immunohistochemical staining for CD34 antigen in human hepatocellular carcinoma. Original magnification for A1–A6, ×200. **(B)** Based on the results of immunohistochemical staining, the HCC patients (n = 165) were divided into high galectin-3 expression (n = 135) and low galectin-3 expression (n = 30) after resection. Log-rank test revealed that patients in the low-expression group exhibited significantly better survival than those in the high-expression group (P < 0.01).

To confirm the prognostic significance of galectin-3 for the early diagnosis and prediction of outcome in HCC, we analyzed the correlation between expression of galectin-3 and clinicopathological and survival parameters. An immunohistochemistry analysis revealed that galectin-3 was abundantly accumulated in the cytoplasm of Hepatocellular cancer cells. We found 135 samples with a high expression of galectin-3 (immunostaining levels of “++” and “+++”) (Figure 2A). The relationship between the clinicopathological features of HCC and galectin-3 expression is summarized in Table 1. We observed that galectin-3 expression was positively correlated with serum levels of AFP (*P* < 0.01). However, there was no apparent relationship between galectin-3 expression and other clinicopathological parameters, including differentiation status (both *P* > 0.05).

**Table 1:** **Relationship between galectin-3 and CD34 expression and clinicopatholo gical features of 165 patients with hepatocellular carcinoma**

**Clinicopathologic variable**	**N**	**Galectin-3 expression**		**P**	**Microvessel counts of CD34**	**Range of CD34**	**P**
		**Low**	**High**				
All cases	165	30 (18.2%)	135 (81.8%)		35.80 ± 20.82	0-77	
Age (years)				0.146			0.952
<50	93	20 (21.5%)	73 (78.5%)		34.64 ± 20.83	1-77	
≥50	72	10 (13.9%)	62 (86.1%)		37.28 ± 20.85	0-76	
Sex				0.353			0.926
Male	133	23 (17.3%)	110 (82.7%)		36.63 ± 20.72	0-77	
Female	32	7 (21.9%)	25 (78.1%)		32.33 ± 21.21	1-74	
Tumor size(cm)				0.393			0.103
<5	67	11 (16.4%)	56 (83.6%)		37.50 ± 19.45	0-77	
≥5	98	19 (19.4%)	79 (80.6%)		34.63 ± 21.72	2-76	
Histological differentiation				0.993			0.217
Good	41	7 (17.1%)	34 (82.9%)		39.89 ± 21.51	3-77	
Moderate	95	17 (17.9%)	78 (82.1%)		33.43 ± 19.48	0-75	
Poor	29	6 (20.7%)	23 (79.3%)		37.74 ± 24.05	7-76	
Liver cirrhosis				0.361			0.621
No	27	6 (22.2%)	21 (77.8%)		37.86 ± 21.43	11-77	
Yes	138	24 (17.4%)	114 (82.6%)		35.39 ± 20.75	0-76	
HBsAg				0.627			0.139
Negative	17	3 (17.6%)	14 (82.4%)		30.61 ± 18.38	1-76	
Positive	148	27 (18.2%)	121 (81.8%)		36.39 ± 21.06	0-77	
Serum alpha fetoprotein				<0.01			0.843
<25 μg/L	47	20 (42.6%)	27 (57.4%)		39.61 ± 20.62	0-77	
≥25 μg/L	118	10 (8.5%)	108 (91.5%)		34.27 ± 20.79	1-76	
Distant metastasis				0.128			0.252
No	152	30 (19.7%)	122 (80.2%)		35.38 ± 20.55	0-77	
Yes	13	0 (0%)	13 (100%)		40.60 ± 24.15	10-76	
Presence of intrahepatic metastasis				0.254			0.82
No	138	23 (16.7%)	115 (83.3%)		34.97 ± 20.73	0-77	
Yes	27	7 (25.9%)	20 (74.1%)		40.03 ± 21.14	3-75	
Recurrence				0.999			0.256
No	154	28 (18.2%)	126 (81.8%)		35.62 ± 20.53	0-77	
Yes	11	2 (18.2%)	9 (81.8%)		38.21 ± 25.54	3-76	

However, high galectin-3 expression was significantly associated with a poor prognosis (Figure 2B). Overall survival was significantly lower in the group with high galectin-3 expression than the group with low galectin-3 expression (135 vs. 30 *P* < 0.01). Further univariate and multivariate analyses were employed to compare the associations of galectin-3 expression with other clinicopathological parameters. Overall survival was significantly correlated with galectin-3 expression (P < 0.01), CD34 (P = 0.049), tumor size (P = 0.005), and histological differentiation (P = 0.032), but not with sex, age, cirrhosis of the liver, serum level of alpha fetoprotein (AFP), serum level of the surface antigen of the hepatitis B virus (HBSAg), metastasis, or recurrence (Table 2). Thus, galectin-3 may be a useful maker for predicting the overall survival of HCC patients.

**Table 2:** **Univariate and multivariate analyses of overall survival in hepatocellular carcinoma**

**Variable**	**Univariate analyses**			**Multivariate analyses**		
	**HR**	**95% CI**	**p**	**HR**	**95% CI**	**p**
CD34	1.01	1.000-1.020	0.049^a^	1.008	0.997–1.018	0.145
Galectin-3	6.228	2.53–15.32	<0.01^a^	7.506	3.000–18.779	<0.01^a^
Age	1.188	0.805–1.752	0.385			
Sex	0.873	0.544–1.399	0.572			
Tumor size	1.808	1.197–2.732	0.005^a^	2.019	1.333–3.058	0.001^a^
Histologic grade	1.408	1.030–1.925	0.032^a^	1.786	1.280–2.493	0.001^a^
Liver cirrhosis	1.007	0.590–1.717	0.98			
HBsAg status	0.963	0.515–1.801	0.907			
Serum	1.24	0.802–1.916	0.333			
Alpha fetoprotein						
Distant metastasis	1.548	0.846–2.833	0.157			

^a^p <0.05.

### Correlation between MVD and clinicopathologic factors in patients with HCC

Intratumoral MVD was quantified by counting CD34-positive endothelial cells in the same series of HCC tissues shown in Figure 2A and the staining intensity of MVD ranged broadly from 0 to 77 micro-vessels/200 × magnification fields. Although a statistically significant difference was not detected, high MVD tended to be associated with histological differentiation (P = 0.217) and tumor size (P = 0.103), and there was no statistically significant correlation between MVD and any other clinicopathologic factors (Table 1). However, MVD showed a significant difference in the low and high galectin-3 group (24.9 ± 18.1 vs. 38.2 ± 20.76, P =0.043). There was a positive correlation between galectin-3 protein and MVD (P = 0.001, R = 0.265).

### Downregulation and overexpression of galectin-3 by using siRNA and Recombinant Lentivirus vector

**Figure 3:**
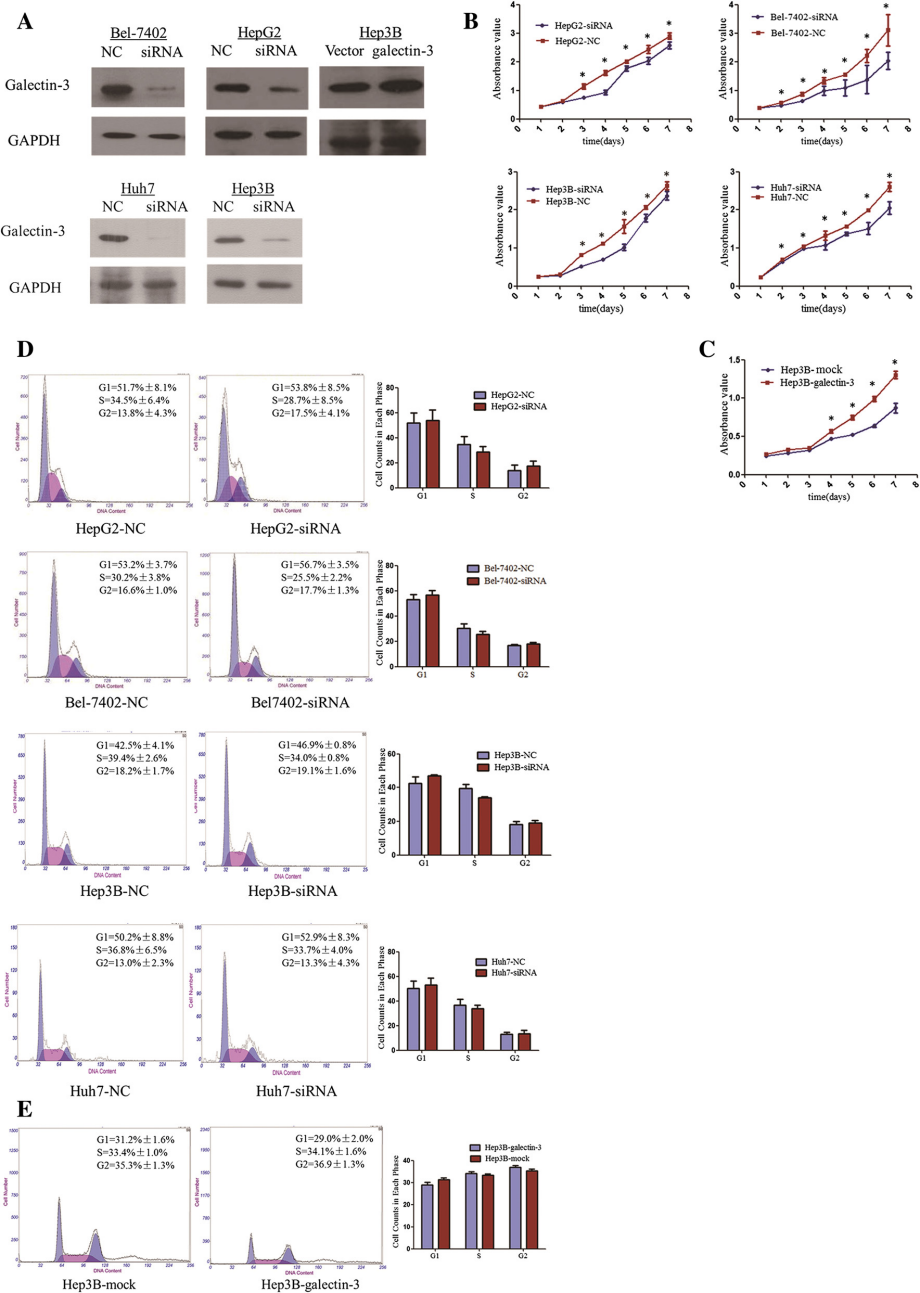
**The effect of galectin-3 knockdown in the viability of HCC cells. (A)** Western blot analysis showing expression of galectin-3 in galectin-3-transfected cells. Expression of GAPDH was used as a loading control. **(B)** The MTS assay showed that silence of galectin-3 inhibited the proliferation of HepG2, Bel-7402, Hep3B and Huh7 cells (*P < 0.05, independent Student’s t-test). **(C)** Rate of cell growth between galectin-3-overexpresion and negative vector -transfected cells by MTT assay. Galectin-3 promoted cell proliferation in Hep3B cells (*P < 0.05, independent Student’s t-test). **(D)** The effect of galectin-3 on cell cycle in HCC cells. The cells were transfected with gal-siRNA or negative control as in D. After 36 hours, the cells were collected for cell cycle distribution analysis. DNA content of gal-siRNA or negative control-transfected cells were detected by flow cytometry. Knockdown of galectin-3 expression did not significantly change the G1, S, or G2 level of HCC cells. Experiments were performed in triplicate times. **(E)** Overexpression of galectin-3 did not significantly change the G1, S, or G2 level of Hep3B cells. Experiments were performed in triplicate times.

To explore the function of galectin-3 in HCC progression, we transfected gal-siRNA and negative control siRNA into four HCC cell lines (HepG2, Bel-7402, Hep3B, Huh7). Silencing efficiency in galectin-3-siRNA transfected cells were verified by Western blotting (Figure 3A). Meanwhile, the Hep3B cells were infected with galectin-3 -vector and control-vector. Galectin-3 expression in the infected cells was confirmed by western blot (Figure 3A).

### The role of galectin-3 in cell proliferation and cycle

To investigate the effect of galectin-3 expression on cell proliferation, we assessed the proliferation rates of control- and gal-siRNA-HepG2, Bel-7402, Huh7 as well as Hep3B cells with a MTS assay. We detected a significant decrease in the proliferation rate of cells transfected with gal-siRNA compared with the rate of the control cells at days 3–7 (*P* < 0.05) (Figure 3B). In contrast, Hep3B cells transfected with overexpression vector significantly increased cell proliferation compared to the control vector (Figure 3C). Furthermore, to ascertain the reason for the restraint in cell growth, the cell-cycle distribution was determined by flow cytometry. The cells were collected for cell cycle distribution analysis after 36 hours, DNA content of gal-siRNA or negative control- transfected cells were detected by flow cytometry. As shown in Figure 3D, silenced galectin-3 expression did not significantly change the G1, S, or G2 levels in HCC cells, including in HepG2, Bel7402, Hep3B and Huh7 cells. This tendency was also found in the Hep3B cells with galectin-3 overexpression vector (Figure 3E).

### The role of Galectin-3 in cell apoptosis

**Figure 4:**
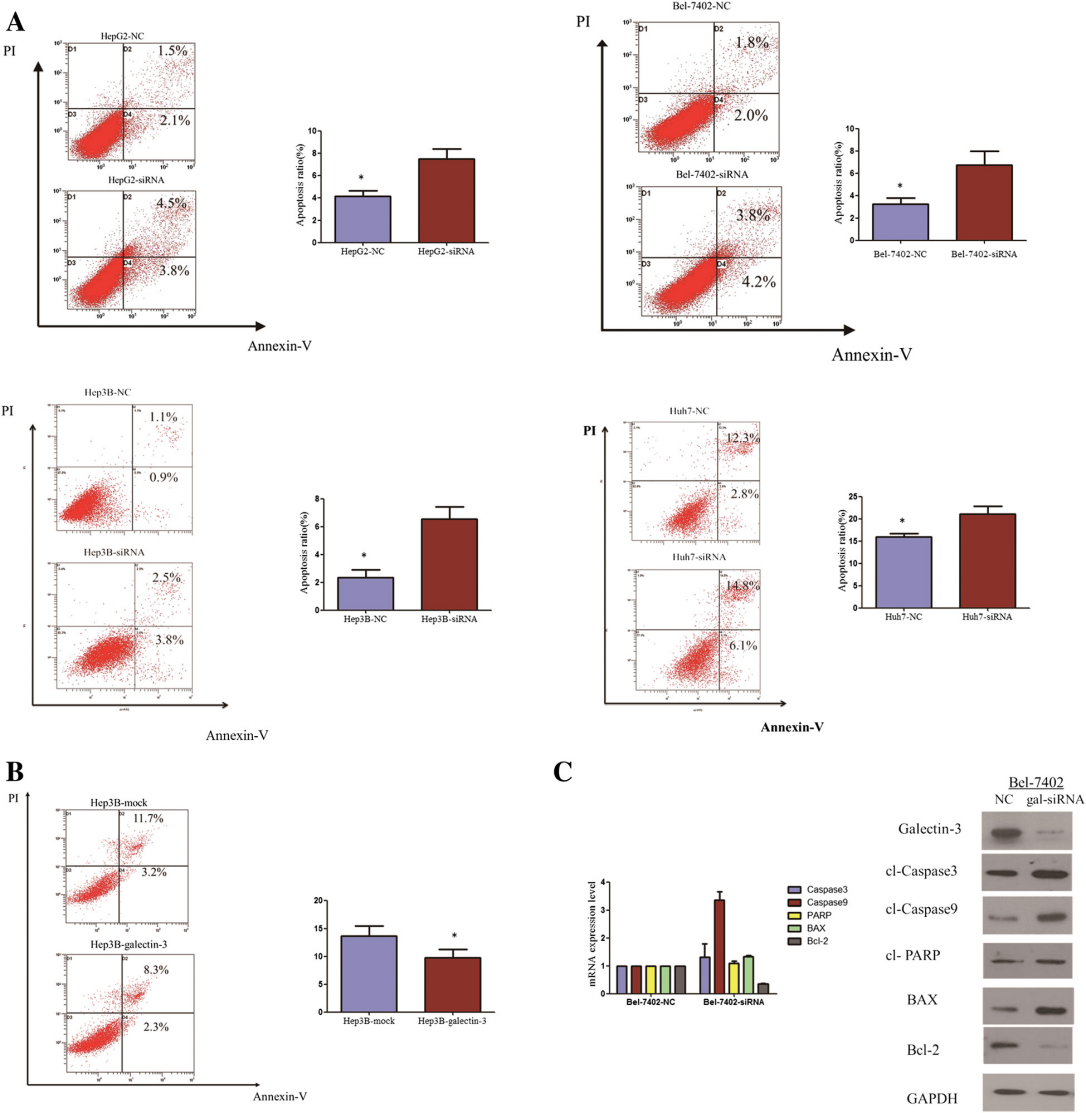
**Galectin-3 knockdown induced the apoptosis of HCC cells, and activates caspase-dependent apoptotic pathway. (A)** Apoptosis levels of gal-siRNA and negative control-transfected HCC cells were quantified with an Annexin V and propidium iodide viability assay. After 72 hours, the cells were collected for apoptosis analysis. The percentages of Annexin-V or propidium iodide-positive cells were detected by flow cytometry. There were statistical differences between gal-siRNA cells and negative control cells. Experiments were performed in triplicate times (*P < 0.05, independent Student’s t-test). **(B)** The galectin-3 overexpression in Hep3B inhibited the apoptosis. Experiments were performed in triplicate times (*P < 0.05, independent Student’s t-test). **(C)** The protein and mRNA levels of PARP, caspase-3/9, BAX, and Bcl-2 proteins were analyzed by western blotting and RT-PCR.

**Figure 5:**
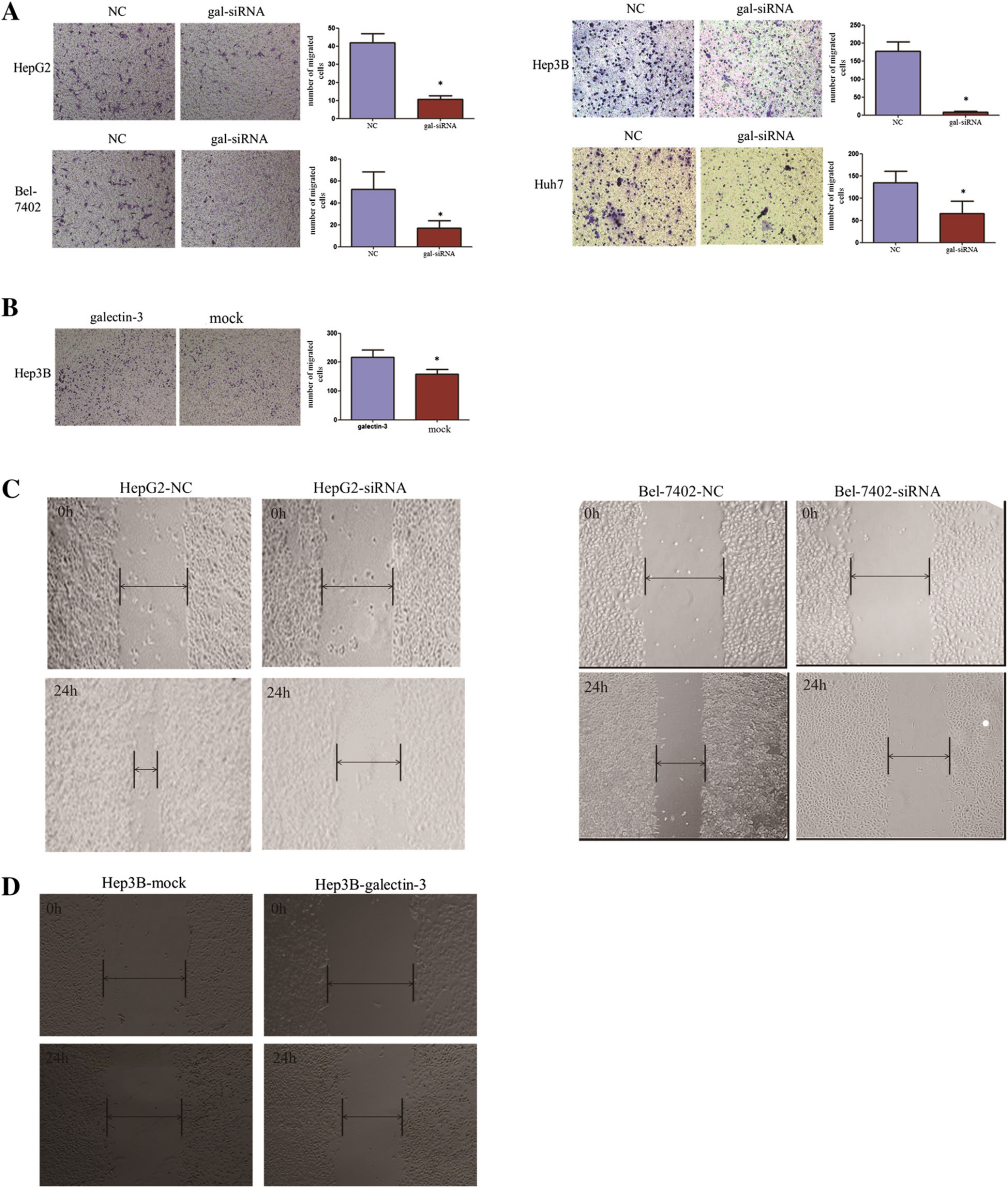
**Enforced expression of galectin-3 promoted HCC cell migration and metastatic capability. (A)** Cell migration of HCC cells transfected with gal-siRNA and negative control was evaluated with transwell migration assays. HCC cells infected with gal-siRNA migrated less than cells infected with negative control. Representative images of migration cells are shown in the left panel and the results are summarized in the right panel. The results are expressed as three independent experiments (*P < 0.05, independent Student’s t-test). **(B)** Transwell migration assay showing that galectin-3 promoted cell migration in Hep3B cells. **(C)** The migration ability of gal-siRNA and negative control-transfected HCC cells was determined by wound-healing assay. Wound-closure time (measured after 24 h) was slower in cells transfected with gal-siRNA than in those transfected with negative control. **(D)** Wound-healing assay showing that galectin-3 promoted cell migration in Hep3B cells. Representative images were taken at 24 hour after scratching.

The next question was whether the effect of galectin-3 knockdown on the inhibition of cell growth was associated with an induction of apoptosis. We then used the Annexin V-FITC binding assay to explore the effects of galectin-3 on apoptosis in HCC cells. After 72 hours, the cells were collected for apoptosis analysis. The percentages of Annexin-V or propidium iodide-positive cells were detected by flow cytometry. Analysis of the proportion of apoptotic cells revealed that a statistical difference between gal-siRNA cells and negative control cells were observed (Figure 4A), Silence of galectin-3 caused an increase in the percentage of apoptotic HCC cells. Furthermore, galectin-3 overexpression resulted in a decrease of apoptosis (Figure 4B). These results suggest that galectin-3 may play an important anti-apoptotic role in HCC development. Caspase activation is an important event in the apoptosis signaling pathway. Thus, to confirm the mechanism of galectin-3 knockdown on apoptosis, we detected the mRNA expression of certain pro-apoptotic proteins and anti-apoptotic proteins, PARP, caspase-3/9, BAX, and Bcl-2 by Real-time PCR. As shown in Figure 5C, galectin-3 knockdown markedly induced the activation of PARP and caspase-3/9, resulting in an increase of the mRNA levels in PARP and caspase-3/9. Galectin-3 knockdown also increased the expression of the pro-apoptotic protein BAX and suppressed the expression of the anti-apoptotic protein Bcl-2. To verify the results observed in real-time PCR, we further examined the protein expression levels by western blotting. In accordance with previous findings, knockdown of galectin-3 markedly induced the activation of caspase-3/9, resulting in an increase in the levels of PARP, caspase-3/9, BAX proteins (Figure 4C).

### The role of Galectin-3 in cell migration and invasion in HCC cells

**Figure 6:**
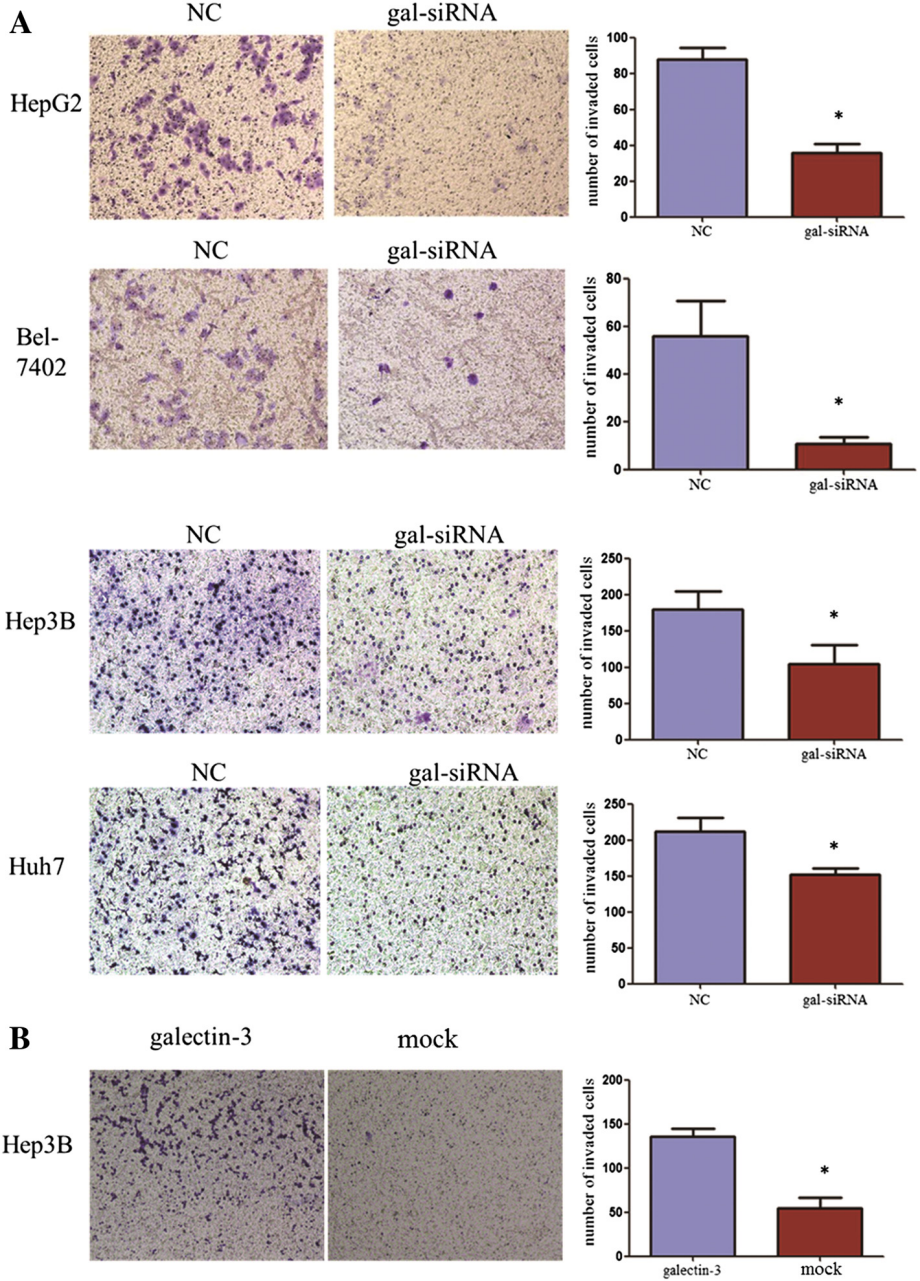
**Galectin-3 overexpression promoted HCC cell invasion, whereas knockdown of galectin-3 suppressed these processes. (A)** Knockdown of galectin-3 significantly inhibited cell invasion in Bel-7402, HepG2 cells, Hep3B and Huh7 cells. Images are shown on the left (magnification: ×100), and the quantification of 10 randomly selected fields is shown on the right. The results are expressed as three independent experiments (*P < 0.05, independent Student t test). **(B)** Galectin-3 promoted cell invasion in Hep3B cells. The results are expressed as three independent experiments (*P < 0.05, independent Student t test).

Previous studies have shown that galectin-3 expression levels are correlated with tumor proliferation in cancer. Hence, we examined the impact of galectin-3 knockdown on the migration and invasion of HepG2, Bel-7402, Hep3B, Huh7 cells. We employed a transwell assay to evaluate the effects of galectin-3 expression on cell migration. HepG2, Bel7402, Hep3B and Huh7 cells infected with gal-siRNA migrated into the lower compartment of the migration chamber significantly less frequently than did cells infected with the negative control (Figure 5A). Wound-healing assays confirmed the inhibitory effect of galectin-3 silence on cell migration. They revealed that the time required for wound closure of galectin-3-silenced HCC cells was significantly longer than that required for the corresponding control cells (Figure 5C).Consistent with the results of the migration assay, galectin-3-siRNA also significantly inhibited cell invasion through a Matrigel-coated membrane (Figure 6A). In addition, we investigated the overexpression of galectin-3 on the migration and invasion of Hep3B. The wound-healing assay and cell migration assay revealed that the ability of cell migration was increased when galectin-3 was overexpressed in Hep3B cells (Figure 5B and D). The invasion results were consistent with the results of the migration assay (Figure 6B).

## Discussion

Galectin-3 is a multifunctional member of the galectin family. It plays an important part in the biological behavior of various tumors and may have diagnostic and prognostic significances [20-22]. Some recent reports have showed an association between galectin-3 expression and HCC. It has been reported that galectin-3 expression is induced in cirrhotic livers and HCC [23] and that galectin-3 overexpression inhibits the immune response by inducing apoptosis in lymphocytes and thus promotes tumor growth [24,25]. Taken together, these results suggest that the expression of galectin-3 has an important influence on the development of malignant tumors. However, the clinical analysis and function of galectin-3 in HCC progression have rarely been discussed in the literature.

In the current study, we examined galectin-3 mRNA and protein expression in 44 paired HCC tumors and adjacent non-tumorous tissues and five HCC cell lines. Most primary HCC tumor tissues showed significantly upregulated galectin-3 expression relative to normal tissues. This tendency was also verified in HCC cell lines. In the immunostaining analyses, increased expression of galectin-3 was found in 81.8% (135/165) of primary HCC samples. The relationship between clinicopathological features and galectin-3 expression showed that galectin-3 expression was positively correlated with serum AFP level (*P* < 0.01), but not with differential stages. Our data are different from those of Shimosegawa et al. [19]. The reasons for these differences may include different genetic backgrounds and a larger study cohort (165 *vs* 52). Immunostaining analyses showed that increased expression of galectin-3 found in 81.8% of primary HCC samples and was associated with serum AFP levels. Furthermore, to evaluate the prognostic value of galectin-3 expression in HCC patients, we divided them into two subgroups (high galectin-3 expression and low galectin-3 expression) and compared outcome between the two groups. The Kaplan–Meier survival analysis revealed that patients with high galectin-3 expression had a significantly shorter survival time than those with low galectin-3 expression. In the multivariate analysis, we observed that galectin-3 expression, together with some traditional prognostic factors (tumor size, histologic grade) were independent risk factors in the prognosis of HCC patients. Similar findings have been reported in other malignancies [26-29].

The biological functions of galectin-3 in HCC are incompletely understood. In the present study, we knocked down and overexpressed the galectin-3 expression respectively, and investigated its effects on the biological behavior. Galectin-3 knockdown in HepG2, Bel-7402, Hep3B and Huh7 cells contributed to inhibit the migration and invasion of cells, which suggested that galectin-3 was associated with metastatic events in HCC cells. Meanwhile, increased galectin-3 expression in the tumor cells stimulates angiogenesis. MVD expression showed a significant difference in the low and high galectin-3 groups. There was a positive correlation between galectin-3 protein and MVD. Proliferation of blood vessels may provide nutrients and pathways for tumor cells and improve its ability of invasion and metastasis, thus affecting the biological behavior of Hepatocellular Carcinoma. This finding is consistent with observations in other human cancers, such as those in the breast, colon, and stomach [30-32].

Increasing evidence has shown that galectin-3 is implicated in the modulation of growth of tumor cells. Galectin-3 contributes to melanoma growth and metastasis via regulation of NFAT1 and autotaxin, and Galectin-3 regulates p21 stability in human prostate cancer cells [33,34]. In the present study, galectin-3 silence in HCC cells reduced cell growth and induced apoptosis. Cell growth differences between gal-siR NA, galectin-3-overexpression cells and control cells in the MTS assay were observed. It has been reported that Galectin-3 silencing inhibits epirubicin-induced ATP binding cassette transporters and activates the mitochondrial apoptosis pathway via β-catenin/GSK-3β modulation in colorectal carcinoma [35]. In this research, we found that the induction of apoptosis in human HCC cancer cells by galectin-3 silence was mediated by caspase-dependent apoptosis pathways. Our results showed that knockdown of galectin-3 induced the activation of caspase proteins. It suggested that the antitumor effect of galectin-3 knockdown in HCC cells is associated with the increased activation of caspase-dependent apoptotic pathway. Margadant C demonstrated that expression of galectin-3 specifically induced by β1 integrins promoted cell adhesion and migration [36]. Zhang et al. found that silencing of the galectin-3 gene inhibited the migration and invasion of human tongue cancer cells in vitro via downregulation of beta-catenin [37]. In the present study, we demonstrated that galectin-3 knockdown slowed the rate of cell migration and decreased the extent of cell invasion, which implies that it may have an oncogenic role in HCC carcinogenesis. However, the underlying mechanism of how galectin-3 activates HCC needs further research.

## Conclusion

In conclusion, we demonstrated that galectin-3 expression levels were increased in HCC, and might be associated with metastasis and cell growth during the progression of HCC. Hence, galectin-3 might serve as a prognostic factor for HCC and have an important role in the diagnosis and treatment of this disease.

## Abbreviations

HCC: Hepatocellular carcinoma
IHC: Immunohistochemistry
MVD: Micro-vessel density
